# A comparison of nasogastric tube insertion by SORT maneuver (sniffing position, NGT orientation, contralateral rotation, and twisting movement) versus neck flexion lateral pressure in critically ill patients admitted to ICU: a prospective randomized clinical trial

**DOI:** 10.1186/s13613-020-00696-2

**Published:** 2020-06-12

**Authors:** Sarvin Sanaie, Negin Mirzalou, Kamran Shadvar, Samad E. J. Golzari, Hassan Soleimanpour, Ali Shamekh, Deepti Bettampadi, Saeid Safiri, Ata Mahmoodpoor

**Affiliations:** 1 Neurosciences Research Center, Aging Research Institute, Tabriz University of Medical Sciences, Tabriz, Iran; 2 Student Research Committee, Tabriz University of Medical Sciences, Tabriz, Iran; 3 Department of Anesthesiology, School of Medicine, Tabriz University of Medical Sciences, Tabriz, Iran; 4 Department of Cancer Epidemiology, Moffitt Cancer Center, Tampa, FL USA; 5 Tuberculosis and Lung Diseases Research Center, Tabriz University of Medical Sciences, Tabriz, Iran; 6 Department of Community Medicine, School of Medicine, Tabriz University of Medical Sciences, Tabriz, Iran; 7 Immunology Research Center, Tabriz University of Medical Sciences, Tabriz, Iran

**Keywords:** Nasogastric tube, Insertion, Complication, ICU

## Abstract

**Background:**

Although many techniques have been introduced to facilitate nasogastric tube (NGT) insertion using anatomic landmarks and a group of devices, there is a lack of general consensus regarding a standard method. The current study purposed to investigate if SORT maneuver (sniffing position, NGT orientation, contralateral rotation, and twisting movement) increases the success rate of NGT correct placement versus neck flexion lateral pressure (NFLP) method.

**Methods:**

A randomized controlled trial study was conducted in two university affiliated intensive care units (tertiary referral center). Three hundred and ninety-six critically ill patients older than 18 years of age were randomly divided into SORT (*n* = 200) and NFLP (*n* = 196) groups. The technique was classified as “failed” after the third unsuccessful attempt. Patient characteristics, success rate for the first attempt, time required for the successful first attempt and overall successful insertion time, various complications including kinking, coiling and bleeding and ease of insertion were noted as main outcomes measured.

**Results:**

Ease of insertion was significantly better in the SORT group compared to the NFLP group (*P *< 0.001). The number of failed attempts was significantly higher in the NFLP group (7.5%) vs the SORT group (3.0%) (*P* = 0.046). The pattern of complications was not different between two study groups (*P *= 0.242). The odds of stage II (odds ratio (OR) = 49.9; 95% confidence interval (CI) 25.2 to 98.6), stage III (OR = 67.1; 95% CI 14.9 to 302.8)) and stage IV (OR = 11.8; 95% CI 3.4 to 41.2) ease of insertion were much higher in NFLP compared to SORT group, after adjusting for age and body mass index (BMI). The odds of failure was not significantly different in NFLP group compared to SORT group (OR = 2.3; 95% CI 0.85 to 6.3), after adjusting for age and BMI.

**Conclusions:**

SORT technique may be considered as a promising method for successful NGT insertions in critically ill patients. However, more trials are needed to confirm the results of this study. The decision must account for individual patient and clinical factors and the operator’s experience and preference.

*Trial registration*: The study was registered at government registry of clinical trials in Iran (http://www.IRCT.ir) (number: IRCT20091012002582N18, 13 March 2018)

## Introduction

Nasogastric tube (NGT) insertion is one of the most commonly performed interventions in critically ill patients [1]. But its insertion in these patients is challenging as they cannot swallow NGT, which results in NGT kinking and coiling in the oral cavity [2]. Furthermore, its flexible nature and presence of an inflated endotracheal tube cuff can make its placement impossible especially during the first attempt [3]. Although its insertion appears simple, due to invasive nature of this procedure, repeated attempts may result in complications like aspiration, intracranial placement, nasal bleeding, esophageal perforation, hydrothorax and empyema [4]. The most common sites of misplacement are piriform sinus, arytenoid cartilage, esophagus and lungs [5–7]. The routine way for NGT insertion is the blind technique with the patient’s neck in neutral position and a lateral neck pressure head flexion. There are several methods which help the insertion of NGT, including reverse Sellick’s maneuver, frozen NGT, with use of endoscope or forceps, stylet, split endotracheal tube and angiography catheter/esophageal guidewire assisted techniques [3, 8–11]. (The list of mentioned methods is fully shown in Appendix Table 4.) Considering feasibility and cost effectiveness, there is growing interest in NGT insertion techniques that are not device-based. It has been noted that most difficulties in NGT insertion are due to anatomic reasons, so to maximize the insertion efficiency and minimize iatrogenic complications, the anatomical variation during NGT insertion must be considered. Najafi introduced a new method named SORT maneuver for NGT placement [12]. SORT is a mnemonic word for the four main steps of the maneuver, namely: **S**niffing position, NGT **O**rientation, contralateral **R**otation, and **T**wisting movement [13]. He recommended that the manoeuver could also be of assistance in trans-esophageal echocardiography probe insertion. Existence of different methods with variable reported success rates indicates that the quest for the best method is still on. The present study was carried out to compare NGT insertion by SORT maneuver with neck flexion lateral pressure (NFLP) in critically ill patients admitted to ICU.

## Methods

This study was a single-blind randomized clinical trial and was registered with the government registry of clinical trials in Iran (http://www.IRCT.ir) under trial number IRCT20091012002582N18. A partial waiver for Health Insurance Portability and Accountability Act (HIPAA) was obtained to allow the investigative team to screen the patients’ charts for their eligibility. An informed consent was obtained from the patients if their cognition level was intact or from the next-of-kin/healthcare proxy if the mentation was suppressed. Five hundred and fifty-one patients who were admitted into two university affiliated ICUs and needed NGT placement were enrolled in this randomized clinical trial.

### Study protocol

**Fig. 1:**
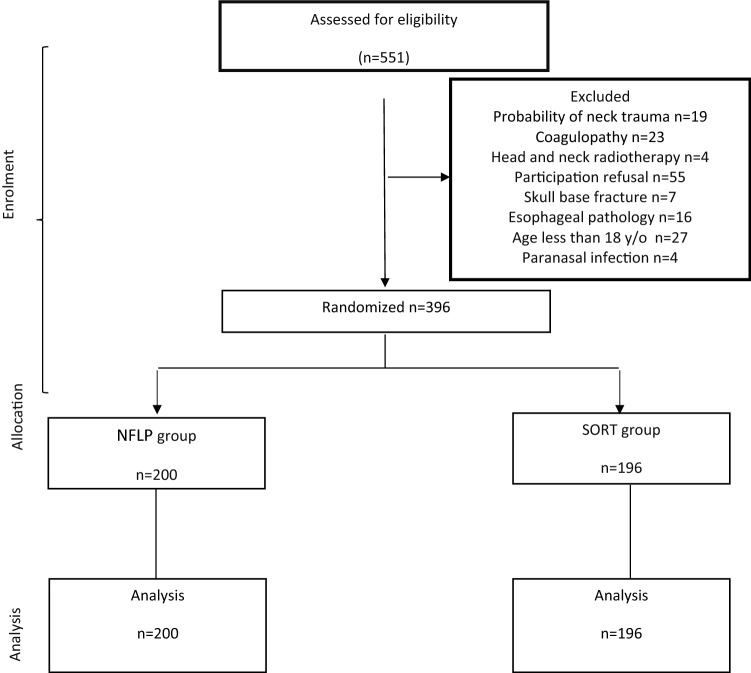
Flow diagram of the study

The study took place at the mixed medical/surgical ICUs of the two main teaching hospitals (1000 inpatient bed) and major trauma centers in Tabriz, Iran from April 2018 to Jan 2019. Flow diagram of the study is shown in Fig. 1. All critically ill patients older than 18 years of age and without skull base fracture, coagulopathy, nasopharynx and esophageal pathology, history of head and neck radiotherapy and neck trauma who needed NGT placement were enrolled in the study. Patients’ refusal to participate in the study was considered as the exclusion criteria. Patients were randomly divided into two groups using a balanced block randomization by the Research Pharmacy team; group NFLP in which NGT was inserted for all patients with standard method (NFLP) and group SORT in which NGT was inserted through SORT maneuver.

A Fr. 14, 105-cm NG tube was used in all cases, and the insertion was performed by two critical care registered nurses who were experienced in NG tube insertion in critically ill patients by these two methods.

As SORT method is a new one and not routine in our ICUs, the nurses who were supposed to perform it had to complete the education course for SORT method. Only two nurses performed the NGT insertion by SORT method in this study in order to decrease interpersonal variations. These two nurses were educated to perform NGT insertion with SORT maneuver for 30 days prior to the start of our trial. They performed almost 35 to 40 NGT insertion with SORT maneuver. We assessed the success/failure rate for them which did not have any difference between the two nurses.

The distal end of the NG tube was lubricated in all cases and passed through the larger nostril to the nasopharynx. The tube was then advanced into the posterior oropharynx according to the selected technique. In Group NFLP, a lubricated NGT was inserted through the selected nostril to a depth of 10 cm. Lateral neck pressure was applied at the same side as that of the selected nostril with the neck flexed and the NGT was advanced to the targeted point. In Group SORT, after the patient was placed in sniffing position, NGT was oriented from the nose to the esophagus entrance considering anatomical landmarks. The position of NGT tip was changed by back and forth and rotational movements until it found its way through the esophagus without any resistance. If any resistance occurred, the procedure was stopped. Sliding distal end of NGT on the posterior wall of oropharynx into the esophagus by the tip of the index finger is sometimes helpful for an accurate orientation. After that, we rotated the head to the contralateral side of NGT entrance. Then, the tip of NGT was directed deep into the esophagus by twisting movements to reduce resistance. We performed external pressure on the area of piriform sinus if the initial maneuver failed. We confirmed the correct place of NGT with epigastric auscultation, aspiration of gastric contents and finally a chest X-ray for reconfirmation. If the first attempt was failed, NGT was withdrawn and fully cleaned and then reinserted in the same nostril. After the third attempt, the technique was considered as “failed” and NGT insertion was guided by laryngoscope and Magill forceps to advance the NGT under direct vision. The NGT length was estimated with measuring the distance from the xiphoid process to the earlobe via the nose [14].

Primary endpoint of our study was success rate for NGT insertion in each group. The secondary end points were complication and ease of insertion in each group.

### Data sampling and recording

Patient characteristics and following data were noted for all patients: success rate at the first attempt, the second attempt and overall for each group, time required for successful first attempt and overall successful insertion time, various complications including kinking, knotting and bleeding. We also reported other rare complications like insertion to cranium, pneumothorax and chylothorax if they occurred.

We evaluated the ease of insertion with a 4-grade score as following: first grade as successful insertion in less than 50 s and in the first attempt, second grade as successful insertion in the first attempt with more than 50 s or in the second attempt with less than 100 s, third grade as successful insertion in the 2nd attempt with more than 100 s or in three attempts, and fourth grade as failed insertion.

### Sample size and statistical analyses

The sample size was calculated based on the pilot study as there was no similar previous study.

A sample size of 168 per group was calculated to identify at least a 10% difference in proportions of failure from the baseline 5% proportion and to fulfill a minimum statistical power of 90% and 95% confidence level. Additional sample size was considered for the multivariable analyses. Finally, 200 and 196 patients were assigned into the NFLP and SORT groups, respectively.

At the first stage, the distributions of quantitative variables were examined using the Kolmogorov–Smirnov test and histogram plots. The quantitative variables with and without normal distribution were reported as mean ± standard deviation and median (interquartile range), respectively. The qualitative variables were reported as frequency (%). Normally distributed variables were compared between two groups using independent samples *t* test and those without normal distribution were compared with Mann–Whitney U test. In addition, Chi-squared and Fisher’s exact tests were used to compare the qualitative variable between two groups. The study variables were compared between the NFLP and SORT groups and those with *P* < 0.1 were further studied whether to be included as confounding variables. Finally, binary and multinomial logistic regressions were applied to examine the association of categorical and binary outcomes with NGT insertion techniques, respectively, after adjusting for confounders. *P* < 0.05 was considered statistically significant. All analyses were done by SPSS software version 19 (SPSS Inc., Chicago, IL, USA) and Stata version 11 (Stata Corporation, College Station, TX, USA).

## Results

Three hundred and ninety-six critically ill patients who required NGT insertion were enrolled in this study. Group NFLP consisted of 200 patients and group SORT consisted of 196 patients. Flow diagram of the study is shown in Fig. 1. Patients in two groups did not have significant differences regarding sex (*P *= 0.370), but the median of age was significantly higher in NFLP than SORT group (*P* = 0.008). Patients in two groups did not have a significant difference in weight (*P* =  0.469), but had a significant difference regarding body mass index (*P* = 0.012). Demographic characteristics of patients are shown in Table 1.

**Table 1:** Demographic characteristics of patients assigned to the study groups

Variable	NFLP (*n* = 200)	SORT (*n* = 196)	*P*
Age (years)	58.5 (24.0)	52.0 (30.0)	0.008
Weight (kg)	70.4 ± 6.0	70.9 ± 7.7	0.469
Sex			0.370
Male	119 (59.5%)	109 (55.1%)	
Female	81 (40.5%)	89 (44.9%)	
BMI	26.9 ± 3.2	26.1 ± 3.3	0.012
Mallampati score			0.621
I	59 (36.4%)	70 (41.9%)	
II	83 (51.2%)	82 (49.1%)	
III	18 (11.1%)	13 (7.8%)	
IV	2 (1.2%)	2 (1.2%)	
Comorbidities			0.820
Diabetes mellitus	65 (32.5%)	60 (30.6%)	
Hypertension	43 (21.5%)	40 (20.4%)	
Ischemic heart dis	63 (31.5%)	59 (30.1%)	
Kidney dis	10 (5.0%)	11 (5.6%)	
Without comorbidity	19 (9.5%)	26 (13.2%)	
Admission type			0.882
Post op	86 (43.0%)	89 (45.4%)	
Trauma	48 (24.0%)	46 (23.5%)	
Medical	66 (33.0%)	61 (31.1%)	
APACHE	22.7 ± 4.5	22.1 ± 4.7	0.195
SOFA	12.3 ± 2.1	12.5 ± 2.4	0.377
RRT	15 (7.6%)	13 (6.5%)	0.669
Vasopressor use	33 (16.83%)	35 (17.5%)	0.860
MV duration	10.85 ± 2.34	11.36 ± 2.71	0.046
ICU LOS	15.34 ± 3.21	15.85 ± 3.62	0.139
Death rate	13 (6.63%)	14 (7.14%)	0.841

NFLP: neck flexion lateral pressure; SORT: sniffing position, NGT orientation, contralateral rotation, and twisting movement; APACHE: acute physiology and chronic health evaluation; SOFA: sequential organ failure assessment; MV duration: mechanical ventilation duration; ICU LOS: intensive care unit length of stay

Before comparing the outcomes of interest between two study groups, the Mallampati score of groups were compared and it was found that the difference between two groups was not statistically different (*P* = 0.621). However, it was found that the pattern of ease of insertion stages were different between the two studied groups (*P* < 0.001). Overall, successful intubation was significantly more in SORT group compared to NFLP (*P* = 0.046). Furthermore, successful insertion in less than 50 s and in the first attempt (Stage I) was more common in the SORT group than NFLP group (89.9% vs. 17.0%). Also, successful insertion in the first attempt with more than 50 s, or in the second attempts with less than 100 s (Stage II) was fewer in SORT than NFLP groups (7.1% vs. 65.0%). In addition, successful insertion in the second attempts with more than 100 s, or in three attempts (Stage III) and failure (Stage IV) were less common in SORT group than NFLP group (1.0% vs. 12.5% and 2.0% vs. 5.5%), respectively. Finally, failed NGT insertion happened in 3% and 7.5% of patients in SORT and NFLP groups, respectively (*P *= 0.046) (Table 2).

**Table 2:** Comparing the outcomes of interest between the study groups

Variables	NFLP	SORT	*P*
NGT insertion			
Successful	185 (92.5%)	192 (97.0%)	0.046
Failed	15 (7.5%)	6 (3.0%)	
Complications			
Absence	128 (64.0%)	136 (68.7%)	
Bleeding	11 (5.5%)	6 (3.0%)	
Kinking	17 (8.5%)	22 (11.0%)	0.242
Coiling	23 (11.5%)	23 (11.6%)	
Multiple	21 (10.5%)	11 (5.6%)	
Ease of insertion			
I	34 (17.0%)	178 (89.9%)	< 0.001
II	130 (65.0%)	14 (7.1%)	
III	25 (12.5%)	2 (1.0%)	
IV	11 (5.5%)	4 (2.0%)	

NFLP: neck flexion lateral pressure; SORT: sniffing position, NGT orientation, contralateral rotation, and twisting movement

Ease of insertion:

I: successful insertion in less than 50 s and in first attempt

II: successful insertion in 1st attempt with more than 50 s, or in 2nd attempts with less than 100 s

III: successful insertion in 2nd attempts with more than 100 s, or in 3 attempts

IV: failure

The pattern of complications such as bleeding, kinking, and coiling or combination of them were not different between study groups (*P* = 0.242). There was not any case of rare complications like insertion to cranium, pneumothorax, chylothorax, etc.

After adjusting for high body mass index and age, the odds of unfavorable outcomes such as Stage II (OR = 49.9; 95% CI 25.2 to 98.6), Stage III (OR = 67.1; 95% CI 14.9 to 302.8) and Stage IV (OR = 11.8; 95% CI 3.4 to 41.2) ease of insertion was much higher in NFLP than SORT group, compared to the reference group (Stage I) (Table 3). Moreover, after adjusting for high body mass index, the odds of failure in the NFLP was higher, but not statistically significant than SORT group (OR = 2.26; 95% CI 0.84 to 6.1).

**Table 3:** Multivariable analysis of the ease of insertion stages and NGT insertion methods

Variables	Odds ratio (95% confidence interval)*			
Ease of insertion	I (reference)	II	III	IV
NFLP vs. SORT		49.9 (25.2 to 98.6)	67.1 (14.9 to 302.8)	11.8 (3.4 to 41.2)

NFLP: neck flexion lateral pressure; SORT: sniffing position, NGT orientation, contralateral rotation, and twisting movement

*Adjusted for age and body mass index

## Discussion

The results of this clinical trial shows that SORT maneuver, as a simple technique, significantly increases the success rate of the first attempt insertion, overall success rate, and ease of NGT insertion, and also decreases the time required for correct NGT placement in critically ill patients admitted to ICU. Although the odds of failure was not statistically different between two groups after adjusting for high body mass index, it was an expected issue as the number of failed cases in two groups, especially in SORT group, was very low and power reduction was not avoidable in the multivariable model. So, this point needs to be interpreted with caution and we should not ignore the lower odds of failure in the SORT group due to the insufficient power of multivariable model.

The insertion of NGT can be difficult even for experienced physicians, as the routine way for its insertion is the blind technique. Variation of patients’ functional anatomy, whether physiologic or pathologic, can furthermore increase the difficulty of NGT insertion.

There are many trials that have used GlideScope or Macintosh laryngoscope with the assisted Magill forceps [5, 8, 15]. But the limited space provided by the laryngoscope or the GlideScope blade for the manipulation of Magill forceps is a drawback of this method which can decrease the success rate or may result in increased complications. Some authors recommended the ipsilateral compression of the neck at the level of the lateral border of the thyrohyoid membrane to transiently collapse the ipsilateral piriform sinus and slightly move the arytenoid cartilage which results in easier insertion of NGT via lateral or posterior hypopharynx [16]. Another technique to overcome the difficulties of blind NGT insertion is considering patients’ anatomical factors. Piriform sinus and arytenoid cartilages are the most common places in which NGT is usually lodged [16].

Najafi introduced a new technique named SORT maneuver for facilitation of NGT placement in anesthetized patients which seems as a suitable approach to solve the mentioned problems [13]. Each component of this maneuver overcomes a problem during NGT insertion. Sniffing position thrusts the arytenoid cartilage away from esophageal entrance. Contralateral rotation of head blunts the ipsilateral piriform sinus malposition while orientation changes the anterior curve of NGT tip to posterior, facing the esophagus. Twisting is for applying back and forth movement to NGT tip in order to reduce resistance during deep insertion until it finds its way through esophagus.

This is the first study that evaluates the effect of SORT maneuver in critically ill patients and our results show that this technique can decrease the failed attempts, the number of attempts and the time required for correct placement of NGT. Kayro et al. showed that using a 5-cm-high pillow was the best way to insert a NGT, but ipsilateral head rotation did not contribute to the procedure [17]. NGT insertion without rotation generally causes impingement of the tip of the NGT on the posterior aspect of the tongue which usually leads to intraoral coiling. The tip of the NGT is always directed anteriorly, so this can also potentially cause misplacement of the NGT into trachea. With SORT maneuver, the tip is always faced posteriorly, hence the tube always advances with the posterior esophageal wall. As a result, it reduces the chances of tube misplacement which is similar to some previous reports [18]. Kinking and coiling of the NGT are the most common complications in previous reports [19], which is consistent with our results.

Nowadays, combined techniques have been considered for NGT placement especially in unconscious patients. Gatack et al. performed a study evaluating the combined facilitating effects of reverse Sellick’s maneuver and neck flexion [9]. In another study, Kirtania et al. showed that esophageal guide wire assisted insertion while maintaining manual forward laryngeal displacement resulted in more successful attempts compared to the technique of head flexion while maintaining lateral neck pressure [7]. Both techniques, like ours, showed the positive results with combining different techniques. One of the most important points regarding correct NGT placement is to develop interventions based on primary caregivers’ knowledge and skills with regard to NGT insertion techniques [20]. This is one of the strengths of our trial as we provided training for our nurses and physicians before starting this trial which may affect the results in the way that produces such a high success rate. This is the first clinical trial regarding efficacy of SORT maneuver in the insertion of NGT in humans and for generalizing the results, we need more trials. Thence, this method may be used as an ideal and simple method for NGT insertion in different situations like TEE as mentioned by Najafi et al. [13].

This study has some limitations. First, this study is not a double-blinded study. Second, we did not enroll children or patients who had not been stabilized. However, the technique should be tested in patients with high risk of NGT insertion difficulty. Although all NGT were inserted by two experienced nurses causing a decrease in the inter-personal variation, we should try this method with different nurses on different populations to generalize the results of this study.

NGT is usually impacted at arytenoid cartilage level and also, inflated balloon of tracheal tube can cause obstruction of the NGT in intubated patients, especially in conditions where cuff pressure measurement is not common. Additionally, cerebral protection is very important in critically ill patients and this technique does not require the use the laryngoscope (that could increase intracranial pressure further). Our four-in-one technique also helps in decreasing the aspiration and ventilator-associated pneumonia chances as this technique does not require deflation of endotracheal tube cuff. We believe that SORT maneuver is a simple technique and does not need any skill for its insertion, but we need more trials to confirm this hypothesis in unskilled inserters. In future, larger studies involving those populations may consolidate the suitability of these modified techniques and may establish the superiority of any of them in the difficult or special situations.

## Conclusion

The SORT maneuver has a high success rate for NGT insertion and increases the ease of insertion. Hence, this method may be considered in critically ill patients, but still there is no consensus regarding a standard approach, and the decision must account for every patient individually and on the basis of clinical factors and the operator’s experience and preference.

## Data Availability

The datasets used and/or analyzed in the present study are available from the corresponding author on reasonable request.

## Appendix

See Table 4.

**Table 4:** Different techniques for NGT insertion

Insertion	
Anatomical	By technique
Lateral pressure on neck	Fibroscopic guided
Cooling of the tube	Endotracheal tube guided placement
Anterior displacement of larynx, lifting of thyroid cartilage	Using Macintosh laryngoscope or GlideScope with assistance of Magill forceps
Neck flexion	Ureteral guidewire
Lateral head positioning	I-gel
Neck flexion lateral pressure	Proseal LMA
Anterior displacement of the mandible (and a group of older techniques)	Ultrasonography
Sort maneuver	Airway scope
	Nelaton
